# Microbes, metagenomes and marine mammals: enabling the next generation of scientist to enter the genomic era

**DOI:** 10.1186/1471-2164-14-600

**Published:** 2013-09-04

**Authors:** Robert Alan Edwards, John Matthew Haggerty, Noriko Cassman, Julia Christine Busch, Kristen Aguinaldo, Sowmya Chinta, Meredith Houle Vaughn, Robert Morey, Timothy T Harkins, Clotilde Teiling, Karin Fredrikson, Elizabeth Ann Dinsdale

**Affiliations:** 1 Computer Sciences Department, San Diego State University, 5500 Campanile Dr., San Diego 92182, CA, USA; 2 Biology Department, San Diego State University, 5500 Campanile Dr., San Diego 92182, CA, USA; 3 Current Address: Scripps Institute of Oceanography, University of California, 9500 Gilman Drive, San Diego 92023, La Jolla, USA; 4 School of Teacher Education, San Diego State University, 5500 Campanile Dr., San Diego 92182, CA, USA; 5 Roche 454 Lifesciences, 15 Commercial Street, Branford 06405, CT, USA; 6 Current Address: Life Technologies, Advanced Application Development, Beverly 01915, MA, USA; 7 Current Address: Immun Array 800, East Leigh Street, Suite 15, Richmond 23219, VA, USA

**Keywords:** Undergraduate education, DNA sequencing, Sea lion, Metagenome

## Abstract

**Background:**

The revolution in DNA sequencing technology continues unabated, and is affecting all aspects of the biological and medical sciences. The training and recruitment of the next generation of researchers who are able to use and exploit the new technology is severely lacking and potentially negatively influencing research and development efforts to advance genome biology. Here we present a cross-disciplinary course that provides undergraduate students with practical experience in running a next generation sequencing instrument through to the analysis and annotation of the generated DNA sequences.

**Results:**

Many labs across world are installing next generation sequencing technology and we show that the undergraduate students produce quality sequence data and were excited to participate in cutting edge research. The students conducted the work flow from DNA extraction, library preparation, running the sequencing instrument, to the extraction and analysis of the data. They sequenced microbes, metagenomes, and a marine mammal, the Californian sea lion, *Zalophus californianus*. The students met sequencing quality controls, had no detectable contamination in the targeted DNA sequences, provided publication quality data, and became part of an international collaboration to investigate carcinomas in carnivores.

**Conclusions:**

Students learned important skills for their future education and career opportunities, and a perceived increase in students’ ability to conduct independent scientific research was measured. DNA sequencing is rapidly expanding in the life sciences. Teaching undergraduates to use the latest technology to sequence genomic DNA ensures they are ready to meet the challenges of the genomic era and allows them to participate in annotating the tree of life.

## Background

The sequencing of the human genome in 2001 marked the beginning of the genomic era [1,2] and since then sequencing technology has undergone major improvements and cost reductions [3,4]. The “next generation of sequencers” enables the sequencing of an ever increasing range of genomes quickly, cheaply and with a high degree of accuracy. Bold sequencing projects, such as the 1,000 bacteria genomes, and the 10,000 vertebrate genomes are revolutionizing life science research and medicine. In medicine, the community is preparing for personal, whole human genomes to become a part of routine care, while a trend to sequence gene panels in human increase until this happens. Even the effects of the human microbial community on human health have been described by DNA sequencing [5-7]. In the environmental sciences, microbes have been identified that are associated with different ecological processes, and the functional profile of microbial communities can be compared across environments [8,9]. In the pharmaceutical industry, sequencing is used in all aspects research and development. Graduates competent in next generation sequencing technologies are needed to support each of these research endeavors, as highlighted in the National Research Council discussion of metagenomics, Clinical Pathologists call to action, and Nature’s discussion on the requisites in genome-jobs [10-12].

While the potential application for genomics is extensive, accelerating our scientific discoveries and simultaneously revolutionizing human lives, the training of the next generation of researchers is lagging [13]. Genomic courses at undergraduate level have been taught at a small number of institutions, however the opportunity for students to gain hands on experience of preparing samples and operating the sequencers is rare. A key aspect in a young scientists’ development is to learn good experimental design practices, which is best achieved by providing experiences across the entire project work flow. In many courses, DNA sequences are obtained from projects available on the web [14] or third party resources, and the students annotate new genes, but do not do any of the sequencing process. Other courses enable the students to extract the DNA, which is sent to a genome center for technicians to sequence [15,16], and the students annotate the new genomes. While annotation has been shown to engage students in analytical thinking, and can allow significant numbers of students to participate in the scientific process [14,17,18] there could be pedagogical and practical value in providing students with opportunities to participate in the whole process, including the sequencing per se. Here we test a new way to engage students, having them work directly with next-generation instrumentation to conduct the DNA sequencing process from the beginning, then annotating the novel genome they sequenced. We invite the scientific community to consider what might be accomplished by the distributed community of undergraduate scientists using this approach.

The most effective way to teach science is to participate in the scientific process [19]. Molecular biology has proven adaptable to educational settings. Cloning projects have allowed students to become technically proficient and learn other important skills of science, such as critical thinking, troubleshooting and adapting protocols to become independent researchers [20]. The development of the “phage hunter” course, where student isolate new phages, obtain sequence data, explore the genomic data, and get to name their phage has been highly successful in training students in scientific discovery and providing new data to science [19,21]. We have built on this excitement of discovery and developed a course that allows undergraduate students to extract, sequence, and analyze novel genomes to become part of sequencing and annotating the tree of life.

The first series of courses in ecological genomics was taught in 2010 at San Diego State University. In the Ecological Metagenomics course, 21 students sequenced novel DNA from microbes, metagenomes and marine mammals. The students were provided with interdisciplinary training in genomics, experience in research, and generated data that is being used by an international consortium to investigate the genomic signature of cancers in the California sea lions. As a template for others to generate next generation DNA sequencing courses, here we describe the ecological metagenomics course, results of student affective surveys, learning outcomes, data quality, and initial findings of the first marine mammal genome sequenced and annotated by undergraduate students.

## Results

### Ecological metagenomics courses

A practical course in DNA sequencing and annotating novel genomes from start to finish with a next-generation sequencer was offered to upper division undergraduates and graduate students as a lecture and laboratory course and was open to students across biology and computer sciences. The syllabus is provided in Additional file 1: Table S1. The goals of the course were to: 1) introduce and use a next generation sequencer and analyse the data, 2) engage the students in research projects sequencing novel genomes, and 3) understand the importance of genomics to areas of biology and ecology. The students were novices in the genomics field as measured by an introductory quiz of the students’ knowledge (Additional file 1: Table S2). None of the students knew when the human genome was sequenced, how much it cost, or how long it took to complete. The students had not been introduced to genomics in earlier classes and had not considered genomics as a research or career area.

**Figure 1:**
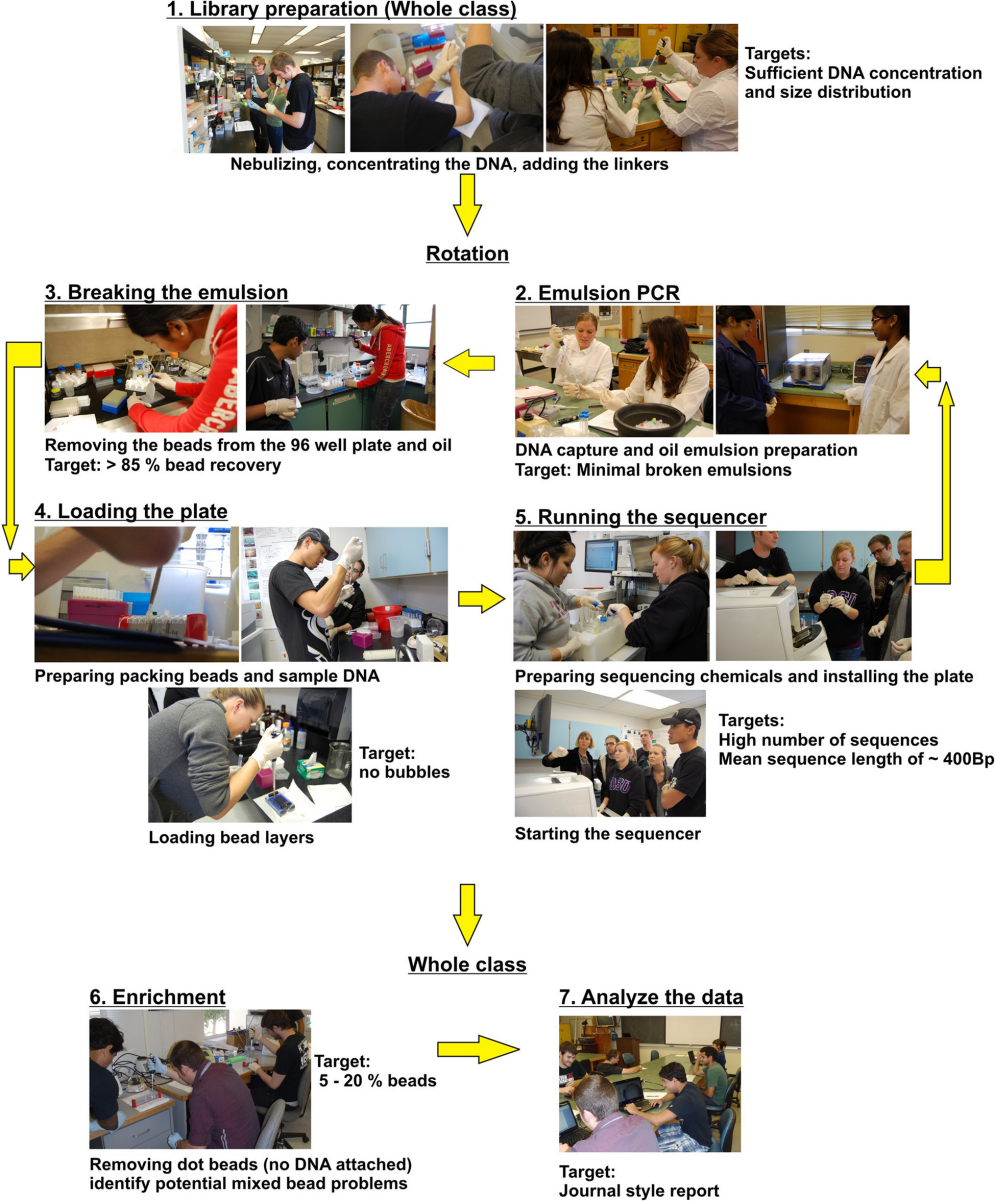
**The seven modules used to teach next generation sequencing to San Diego State University students.** The first module is taught to the whole class and the next four modules are taught on a rotation basis, with each student taking part in a different piece of the process and then rotation the following week. Module 6 and 7 are taught to the whole class.

The course was taught using a 454 FLX titanium sequencer, and covered the entire DNA sequencing process. Each process has multiple steps and requires students to follow complex protocols and carefully conduct the steps in a time sensitive manner. Conducted by one person the process takes about 3 days. To fit all of the components into the course, in which the students were only in the lab for 3 hours one day a week and ensure that the students had practice in all steps; the process was redesigned to seven modules that were taught on a rotational basis. Similar adaptations could be made for all sequencing platforms. Therefore, every student played a part in every sequence run, the sequencer was run most weeks, and sequences were ready for analysis early in the semester (Figure 1).

The course focused on sequencing the California sea lion (*Zalophus californianus*) and the microbial communities from the kelp forest where the sea lion hunts. The different organisms piqued each student’s interest, and the range of genome sizes provided different characteristics that were useful in practical conduct of the course. For example, the large genomes provided a control sample that could be sequenced multiple times at the same titration level throughout the course, facilitating the students’ introduction to the sequencer, and providing the instructor a tool to evaluate each student’s progress. The microbial samples allowed the students to culture organisms and extract DNA for sequencing. Because each microbial genome library was different, the students were required to calculate the titration level for each library. By calculating the titration levels (i.e. the amount of DNA per capture bead that is required for a successful sequencing run), the students understood the effect of varying DNA quantity on the sequencing quality.

Several metrics were used to determine the success of the ecological metagenomics course, including quality of sequences data, course evaluation, student self-confidence, and student learning. The quality of the data produced by the students was assessed using the quality control targets set by the manufacture, sequencing number and average length, and contamination levels. Course evaluation was administered at the end to assess whether the students had perceived that the course had met its education goals. Self-confidence and learning surveys were administered at the beginning and end of the course. Student learning was measured by changes in responses to 10 open-ended questions (Additional file 1: Table S2). Changes in students’ self-confidence in their ability to sequence DNA and to conduct scientific research were measured using scales adapted for this particular course [22,23]. Both scales were found to be reliable (Cronbach’s α = 0.96, 0.92) and changes were measured using a matched-pairs *t*-test.

### The California sea lion genome case study

The California sea lion, *Zalophus californianus*, is a coastal sea lion that ranges from the west coast of southern Alaska to the Baja peninsula in Mexico [24]. The California sea lion population is growing and while iconic for the California students, they are often in conflict with humans because they exploit prized fisheries such as swordfish and salmon [25]. The California sea lion is most closely related to the Galapagos sea lion and the extinct Japanese sea lion [26]. Sequencing mammal genomes provides information on evolution and identifies genes that are responsible for specific traits, in this case the return of a land mammal to a semi-aquatic habitat. Many mammalian diseases have a genetic component and identifying linages specific genomic changes may shed light on defects in related organisms. Understanding structural and functional features that influence genome size and evolution may be important in ecological and population studies designed to address issues relating to coastal conservation. The California sea lion is the first marine mammal genome, the first from the suborder Pinnipedia, and the fourth carnivore to be sequenced. The assembled DNA totals 1,951,532,210 bp with 13,352,265 bp in contigs > 10 kb, and 972,007 bp in contigs > 15 kb. The N50 sizes are 2,127 bp for all contigs, 11,249 for the 10 kb contigs, and 16,472 for the 15 kb contigs, suggesting high quality sequencing. The sea lion data is available from http://www.sealiongenome.org upon request and will be released after publication from NCBI.

**Figure 2:**
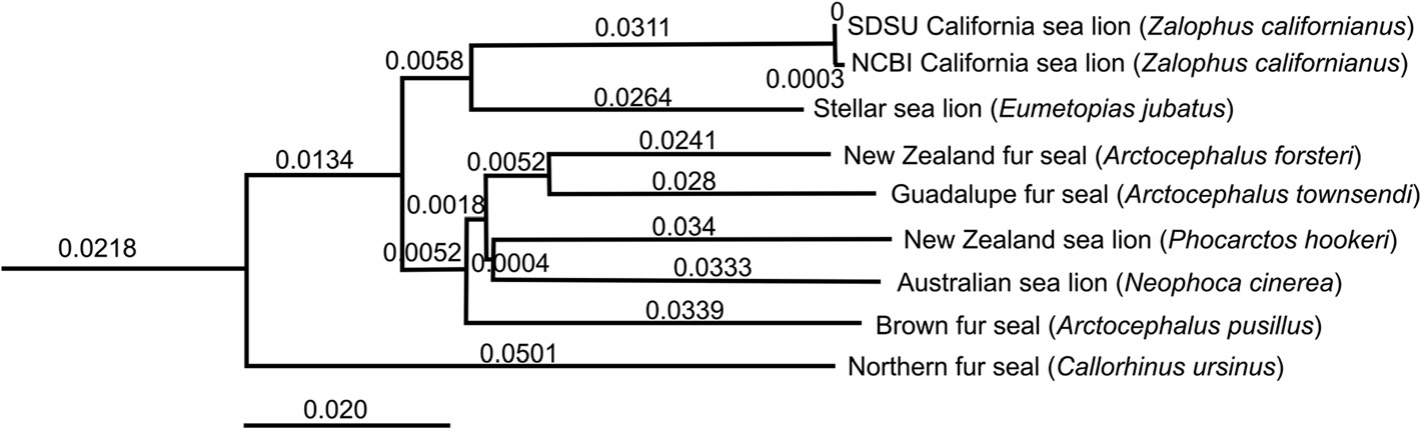
**Phylogenetic tree of the mitochondria California sea lion sequenced by the SDSU students compared with the sequences in NCBI from a range of Pinnipedia.** The branch length between SDSU mitochondrial genome and NCBI mitochondrial genome was observed to be 0.0003, suggesting they were nearly identical.

The sea lion genome is 34.7% repeats (Additional file 1: Table S3), which is consistent with carnivores, including the dog (30.4%) [27] and the panda (36.7%) [28]. The level of repeats is ~ 10% less than the human genome [29]. The repeat regions were dominated by the LINE1 category, similar to other carnivores. In contrast Alu repeat sequences, which are abundant in the primate linage [30,31], were not present in the student sea lion genome, showing minimal human DNA contamination of the sequences. The mitochondria DNA was completely sequenced and showed a 100% sequence identity to California sea lion mitochondria from NCBI accession number AM181017.1. The phylogenetic comparison of the mitochondria placed it within a group containing other sea lions and fur seals (Figure 2).

### Course outcomes

**Figure 3:**
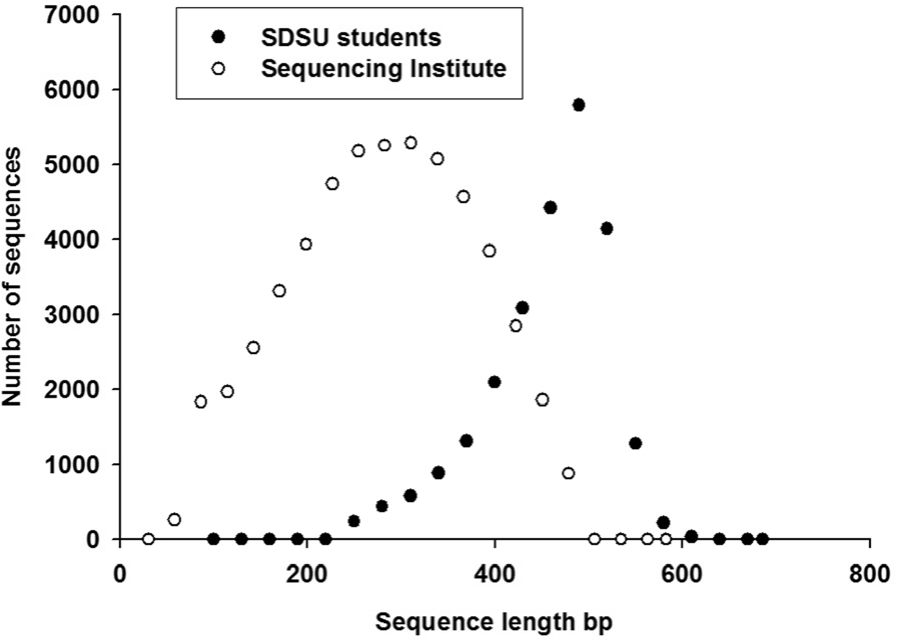
**Comparison of sequence length and quantity of a random selected sequencing run from the SDSU students and a run conducted by a sequencing institute.** The DNA that was sequenced in each run is from different microbial organisms.

To assess the outcomes of the course, we examined the quality of student data, course evaluations, changes in students’ self-confidence, and student learning. At the end of the course (15 weeks), the students had sequenced 14 bacterial genomes, 14 metagenomes and approximately 5x coverage of the sea lion genome. The students met the manufacturers recommended key quality targets for the sequencing process. Targets include correctly sized libraries (> 7.3 x 10^8^ molecules of DNA with peak of the DNA sample migrating to between 500 and 1,250 bp), bead recovery (>85%) and enrichment levels (between 5-20%). Each run of the sequencer should provide approximately 800,000 to a million sequences. The number of sequences will depend on the DNA quality and the operation of the sequencer. There are various filters built into the sequencing software, these include removal of short sequences and those of poor quality. Therefore, a successful run should have about 2 million beads loaded onto the plate (identified using the key pass process) and a good quality run will retain ~ 50% of these sequences, which was shown in the three randomly chosen students’ runs (Additional file 1: Table S4). To analyse the quality of the data, the length of the sequence can be assessed. The sequences on a single run will range in length with a tight peak of sequences around the average length for the individual sequencer. The length of sequences obtained from a plate sequenced by the students was compared to one from a professional lab and it shows the students had a peak of sequences around the 500 bp long, with few short sequences (Figure 3).

Human contamination is a potential problem with novice users. Human contamination is difficult to discern in eukaryotic DNA, because there is an over whelming bias of human DNA sequences in the databases. Therefore, the amount of contamination in the metagenomics samples was calculated using BLAST. A metagenome is a random sample taken from a microbial community and contains short sequences that are generated from different microbial taxa and different genes [9]. The metagenomes are microbial and therefore should contain very few sequences similar to human DNA (some sequence show similarity to human genes because of evolutionary history and the bias towards human sequences in the databases and some metagenomes may contain eukaryotic sequences). As shown in Additional file 1: Table S5, almost no human DNA matches were found suggesting the students were not sequencing themselves. Another test of the quality of the sequences generated by the students would be to see whether the proportion of sequences in the metagenomes that show similarity to microbes or functional genes were similar to those described in the metagenomes from the literature. The microbial communities sequenced by the students had between 37–76% of the sequences that showed similarity to various Bacteria and Archaea and 24 – 53% of sequences similar to known functional genes, similar to that of an externally sequenced metagenome (Additional file 1: Table S5). The proportions sequences similar to known organisms in the student sequenced metagenomes is similar to those describes for other marine samples in the literature [9,32,33], further suggesting that the student were generating usable sequence data.

The students’ final project was a formal report where they described the characteristic of the genomes, specific metabolic pathways or suggested how the features of genome contribute to the activity of the organism. The students investigated viral, bacterial, archaeal and eukaryotic genomes using 660 billion bp of sequence data and some of their project titles, amount of sequence examined and brief findings of the students is shown in Additional file 1: Table S6. Several of the ecological student reports are in the final stages of manuscript preparation for submission to peer reviewed journals and sequences generated by the class have contributed to two publications [34,35] and two new genome descriptions [36,37].

**Figure 4:**
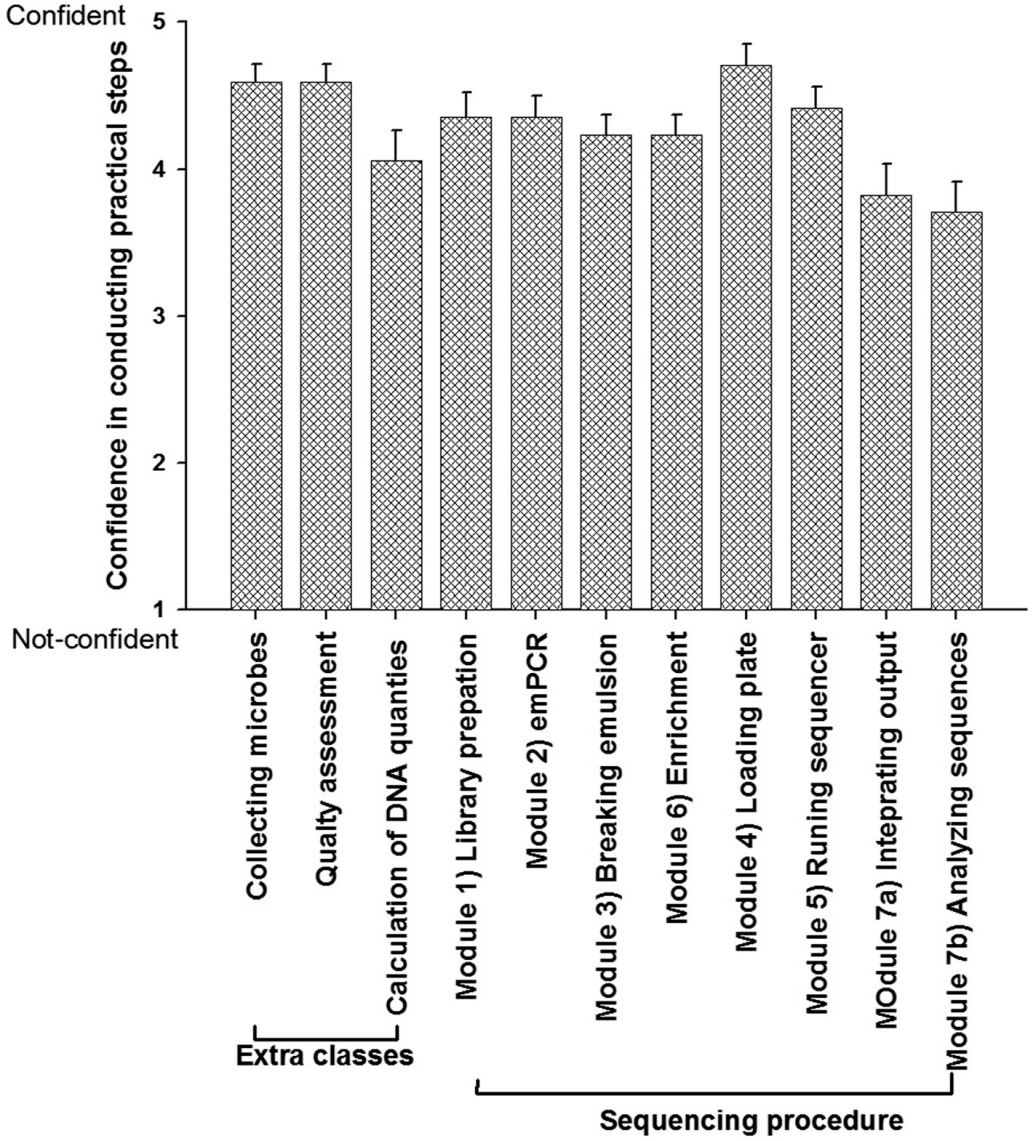
**The student’s perceived ability to conduct each module of the DNA sequencing course.** The students felt confident in conducting the practical side of the course, but were less confident in the analysis. To address the lower confidence in the analysis area, we provide an ecological bioinformatics course that provides an in depth analysis of genomic data in subsequent semesters.

**Figure 5:**
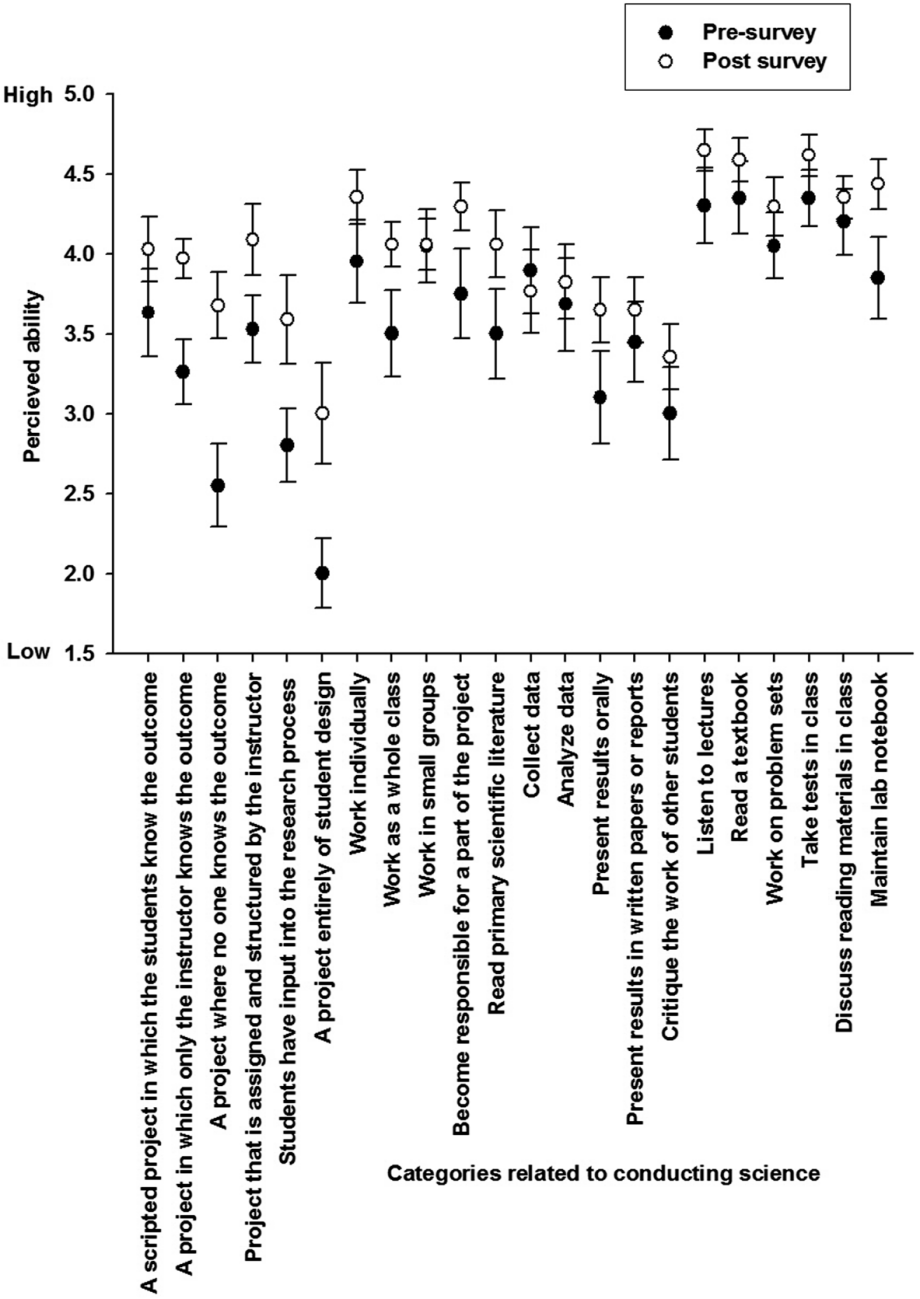
**The student’s perceived ability to conduct independent research.** Significant gains were shown in the abilities of students to conducting projects where no one knows the outcome of the research and were they responsible for part of the project.

The students (n = 19) evaluated the ecological metagenomics course relative to its goals of; 1) to introduce and use a next generation sequencer and analyse the data, 2) to engage the students in research projects sequencing novel genomes, and 3) to understand the importance of genomics to areas of biology and ecology) as 4.7 (±0.1) out of 5. A mean rating of 4.3 (±0.2) (out of 5) was given to the students’ perceived confidence in conducting the 7 modules described in Figure 1, lower scores were given to the analysis section (Figure 4). A major objective of the ecological genomics series is to increase student ability to conduct research. On two different measures assessed, there was a significant increase in scientific competency. Students’ self-confidence in their ability to conduct DNA sequencing (n = 18/21) increased from 3.0 (pre) to 3.9 (post) (t = −3.21; p < 0.01). Students’ perceived confidence to conduct scientific research (n = 19/21) increased 3.3 (pre) to 3.8 (post) test (t = −2.15; p < .05). The students showed an increased confidence in conducting projects where 1) no one knows the outcome, 2) they have input into the process, 3) they need to work as a whole class and 4) they have responsibility for part of the process (Figure 5). The students increased in their ability to interpret primary literature, present data and keep a lab book. Skills required in becoming a successful scientist. The students did not vary in the ability to listen to lectures, take notes, read a textbook or work on set problems, all skills they learned in traditional courses, but these activities were not a focus of this capstone course. The change in the student perceptions match the types of activities that were conducted in class and therefore the class was highly successful. All students would recommend the course to other students and had extremely positive comments about the course and some are listed in Table 1. Students increased their knowledge about genomics by discussing journal articles, analysing the large amounts of data, and writing a formal report about their data. The students’ scores (n = 21) on the pre and post quiz almost doubled from 2.6 (+ 0.29) at the beginning of the class to 4.26 (+ 0.15) at the end of the class (questions provided in Additional file 1: Table S2). For example, students were unable to answer question 6, “Describe how pyrosequencing works” at the beginning of the class but provided at least a ½ page answer at the end of the course. In addition to the sequencing concepts, at the beginning of the course the students did not know about the activity of microbes and viruses in the environment and afterwards they were able to provide detailed descriptions of metagenomics and the information that was obtained by sequencing environmental microbes and viruses.

**Table 1:** Comments provided by the students describing their thoughts about the ecological metagenomics course

**Student**	**Comment**
1	This next generation sequencing experience has been educational beyond any class I have taken. It would be a mistake if this class were terminated. It would be a mistake if Dr Dinsdale was not given props for organizing this class. She needs to teach it again – it was fun!
2	I thought this class was very interesting and needs better advertising. I got into the course by accident but the quality of the course deserves more student interest. Being an ecology guy, I would have liked to have more background on how these microbial communities can affect the larger environment.
3	This was a very exciting course that introduced what I think is the next big thing in science. Being able to sequence essentially on demand is going to enhance a lot of research. It was a lot of stuff to take in.
4	This was one of my favourite courses I’ve ever taken at SDSU. I feel like I’ve learned so much and this class has spawned my interest in genomics. I am really excited about the new technology this class offers and I would highly recommend this course to others. I am really glad I had the opportunity to take this course.
5	As a student, I feel that doing the labs I was given enough independence to feel I was doing the work on my own and with my lab mates. This is a very important part of a lab course and I believe it should be preserved.
6	I liked the course and learned a lot. I feel confident about the Next Generation Sequencing, but would suggest more time reading and understanding the flow grams and analysing the data.
7	This course has been very useful to me with every aspect of sequencing touched and explained.
8	Overall, it was a good course especially to me, who didn’t have any lab-experience. It taught me the lab side of the sequencing, the process and the chemistry involved.
9	I loved the class. I think designating 10/15 min at the end of the lecture to talk about what’s going on in lab for the week would have been useful, that way we can come to lab feeling more prepared.
10	This was a great course to take at SDSU and I am grateful for the opportunity to be one of the small numbers of students to take this course. All labs were hands on and very educational.
11	Excellent course! This course has opened doors for me in the industry! I have got 2 calls and had an interview by saying that I’ve taken the course.

## Discussion

In a time when education and research are suffering budgetary constraints, introducing a sequencing based course into undergraduate training was high risk, but has returned high rewards. Publishable quality data has been generated and the students were provided with state of the art training. New technology engages students [38], and the genomics course merges the new technologies of metagenomics and next generation sequencing. While the sequencing technology is changing rapidly, by conducting the process on one instrument the students will be able to understand the new developments and the gains that the students made in terms of thinking like a scientists will last them a lifetime. The course inspired students to follow a genomic career path and several are employed in related industries or continued their education in the genomic arena. Career pathways that are not only highly relevant in today’s society, but ones they had not considered prior to taking the course. The students have gained knowledge and skills that are not offered in traditional lecture- and laboratory-based course which follow a cook book approach. Instead, these students are engaged in real research, and generating data that is useful to researchers across the world. Data will be released through SEED, MG_RAST and NCBI upon publication.

The course is cross-disciplinary, bringing together biologists and computer science students. In genomics, bioinformatics and analysis of the resultant data has now become the bottleneck of most sequencing projects. Part of the problem arises from a lack of training in both biology and computer science. This course has the two groups of students working side by side, the computer scientists learned biology and the biologists learn some of the computational constraints and both groups of students learned research techniques. Enabling collaboration of these students at an early stage will help the progress of bioinformatics in the future.

The lab is costly, time and instructor intensive, but the reward are large as it provides students with research experience in a technology of the future and acts as a recruitment tool for the life sciences. There are several problems inherent in teaching students DNA sequencing, 1) the potential of human contamination, 2) contamination of the environmental DNA with linkers or other cross-contamination of samples, 3) damage to the equipment with inexperienced researchers, and 4) establishing metrics to enable the assessment of good laboratory practices. Using basic laboratory sterile techniques successfully limited contamination issues. Setting the course up on a rotational basis by having students working in different rooms and leaving after each step stopped cross-contamination with linkers. The students were closely guided when operating the sequencer and recognized the opportunity, thus respected the equipment and no damage to the sequencer occurred during the course. By dividing the protocols up into lab timed blocks, the whole sequencing process takes longer than would be recommended by the manufacturer, but the time lag did not lead to noticeable reduction in yield. The time lag made it difficult to respond to any sequencing issues, such as over or under enrichment. Keeping track of each part of the process being conducted by each group of student was initially difficult and therefore a new online database that could be accessed by students and instructors was developed and would be available to other researchers on request. As with any course, pedagogical goals were reinforced by repeatedly covering material and using assessments to reinforce learning outcomes.

## Conclusions

DNA sequencing is one of the fastest growing fields in the life sciences; however students have problems relating to the concepts because of the complexities, amounts of data, cross-disciplinary and microscopic nature of the process [39]. By providing students with the opportunity to use a sequencer and sequence novel organisms, some of the mystery of sequencing was removed and the students were motivated to explore the complex data. Students attended capstone courses, became part of many research projects, including an international consortium, and were provided training to enter the genomic era. Many students have continued their scientific careers in either the academic or industry side of the business, suggesting the power of DNA sequencing to recruit much needed talent to the life sciences and extend the capacity and use of DNA sequencing. The best summary of the course comes from the students “This course is a ‘must have’ in the resume of any molecular biologists, graduate or undergraduate. The technology we were able to use and the research projects we have been part of constitute an unbelievable asset that without any doubt will be very useful in our professional futures”.

## Methods

### Teaching procedure

To conduct a sequencing run on a 454 FLX titanium sequencer takes a single person approximately 3 days, but the procedure needs to be divided into 2 hour: 40 minute modules and be conducted by 20+ students. Therefore, to organize the course to maximize equipment and learning objectives, some modules of the class were taught to all students at once and other parts of the course were taught to groups of students on a rotational basis (Figure 1). Written consent was obtained from the students to display their photographs. The rotation allowed the students to be in small groups and obtain practice in all areas of the process. The sequencer could be run on a weekly basic, ensuring sequences were available for students to analyse. The 454 protocol was broken up into seven modules (Figure 1), 1) library preparation, 2) emPCR, 3) breaking the emulsion, 4) loading the beads into the picotitre plate, 5) running the sequencer, 6) enrichment and 7) analysing the data. Module 6 - enrichment, is out of order, because is too lengthy to put into the class schedule more than once, therefore, for most of the semester a teaching assistant does this step in the rotation. Three lab sections were devoted to analysis of the data and the students had a further two weeks to finish their reports (Additional file 1: Table S1). Some extra classes that could be included to round out student education are collecting microbes, extracting DNA and quantification of the DNA. The extra classes provide students practice at several of the techniques prior to working with the 454 sequencing chemicals. For the whole class modules, the students worked in pairs and in the rotation modules, the students worked groups of four and often each module were subdivided such that the students worked on half of the protocol and then brought their products together at the end to complete the section. For example, in the emPCR module, a pair of students would organize the DNA capture and the second pair would prepare the emulsion oil and at the end, the DNA would be combined into the oil and both groups would pipette the oil into the PCR plates. Each module was conducted several times during the semester to enable each student to conduct each part of the process. The 454 was always run with the picotitre plate divided into four lanes, thereby giving the students more practice at the various steps. Each step had important targets that the students had to meet as part of their grades. For example, in the library preparation the amount of DNA in the library and the length of the fragments were measured using a bioanalyzer (Agilent 2100) and these need to meet the manufactures requirements (> 7.3 x 10^8^ molecules of DNA with peak of the DNA sample migrating to between 500 and 1,250 bp). A 2 hours lecture section was held in conjunction with the practical course and provided theoretical background for understanding the sequencing technology and analysing the data. The lectures were divided into four sections that described; 1) next generation sequencing, 2) metagenomics, 3) eukaryotic genomes and 4) bacterial and archaeal genomes. The lectures relied on journal articles that were presented by both the professor and students (Additional file 1: Table S1). The presentation format increased student participation and provided examples of how to analyse the sequence data, which students would need to use in their final report.

### Sequencing the California sea lion

DNA from a male sea lion was provided by Y. Schramm and G. Heckel. DNA was cleaned using high template PCR cleaning kit (Roche), and 70 μg of DNA was obtained. The students sequenced the sea lion genome in the courses held in Spring 2010–2012. Sequences with homology to the mitochondria were identified by comparison to a local version of the mitochondrial sequence database (http://megasun.bch.umontreal.ca/ogmp/projects/other/mtcomp.html) and separated prior to assembly. The mitochondrial sequences and the remaining (chromosomal sequences) were assembled independently using Newbler version 2.6 (454/Roche Life Sciences, Branford, CT). Sequences related to the mitochondrial genome were identified by BLASTN at the NCBI website and similar sequences were downloaded and aligned using ClustalX [40]. A distance matrix was computed from the alignment using phylip [41] and visualized using FigTree (http://tree.bio.ed.ac.uk/software/figtree/). Mitochondrial genome alignments were also compared using Mauve [42]. Interspersed repeats and low complexity sequences were identified using RepeatMasker v. 2.3.8 [43]. This program also provided GC skew information. These programs were run by the students.

### Preparation and analysis of microbial genomes

Marine microbes were obtained by the students by plating 100 μl of seawater on three different growth media: TCBS, MacConkey, and Marine Broth with 15 g/l agar added. All cultures were incubated at room temperature overnight and individual colonies were re-streaked until a single colony was obtained. Bacterial DNA was extracted from 1 ml of a liquid overnight marine broth cultures inoculated with a single colony. The overnight culture was pelleted and re-suspended in 600 μl of nuclei lysis buffer and incubated at 80°C for 5 min. The samples were cooled to room temperature and 200 μl of protein precipitate solution was added. The mixture was vortexed at high speed for 20 s and incubated on ice for 5 min. After centrifugation (13,000-16,000 x g, 10 min), the supernatant containing the DNA was transferred into 600 μl of room temperature isopropanol. The tubes were gently inverted until thread-like strands of DNA were visible. The DNA was pelleted (13,000-16,000 x g, 5 min) and washed with 70% room temperature ethanol, centrifuged again, and was air-dried overnight then rehydrated in 100 μl of ultrapure water. Microbial genomes were sequenced of a quarter of plate of 454 flex and assembled using Newbler version 2.6 (454/Roche Life Sciences, Branford, CT). The assembled genomes were up loaded to the RAST and annotated using subsystem technology [44]. The students examined each genome and obtained the required data for their reports.

### Preparation and analysis of the metagenomes

Metagenomes were prepared by concentrating approximately 60 l of seawater using a tangential flow filter (tff). A demonstration of the tff was provided to the class, because the concentration process allows the students to see the microbes. Once concentrated the microbes were obtained by filtering through a 0.2 um sterivex. The DNA was extracted using phenol chloroform extractions [9,32,33]. Because of the long lag time in the metagenomic DNA preparation, this part was conducted by the class teaching assistant. The metagenomes were analyzed without assembly using MG-RAST [45,46]. Sequence similarity was set at an e value of 10^-5^, percent identity of 60% [9,32,33]. The students examined and compared metagenomes within the MG-RAST platform for their reports.

### Evaluation of student learning outcome and ability to conduct STEMS research

An evaluation was conducted on the course to identify whether the students felt the course had reached its goals. The students were asked whether they were confident in conducting each module described in Figure 1 of the sequencing process, annotating the data and whether they would recommend the course to other students. The evaluation was conducted on a likert scale from 1 to 5, where 5 indicated that the students highly agreed with the statement and 1 indicated that the students highly disagreed with the statement. In order to assess the impact of the ecological metagenomics course, we administered an affective survey to measure students’ interest in and understanding of their field of genomics. The survey instrument measured students’ confidence in their ability to:

(1) sequence DNA [23] and

(2) conduct science research [22]

These surveys were adapted to meet the needs of our specific course and the revised versions were all found to be reliable (Cronbach’s α = 0.96, 0.92, respectively). These measures were administered and at the beginning and end of the courses. The study was presented to the 21 students that took the 2011 ecological metagenomics course and 19 participated in the survey. Paired samples test was used to identify significant in the pre- and post- surveys.

## Competing interest

The authors declare that they have no competing interests.

## Authors’ contributions

RAE, EAD developed the course, analyzed the data and wrote the manuscript; MHV helped develop the assessment instruments and analyzed the perceptual data; JMH, NC, JCB, KA were teaching assistants on the course; TTH, CT, KF provided technical support for the course; SC and RM provided analysis of the sea lion DNA. All authors read and approved the final manuscript.

## Supplementary Material

Additional file 1: Table S1 Lecture and lab schedule for the ecological metagenomics class. **Table S2.** The question for the Pre and Post quiz given to students in the ecological metagenomics class. **Table S3.** The proportion of repeat regions identified in the California sea lion, panda, dog, human, and mouse. **Table S4.** The number of sequences that met each of the filter controls on three sequencing runs conducted by the students on a titanium plate divided into 4 lanes. **Table S5.** The sequence characteristics of three metagenomes, constructed from the surface water off Mission Beach (California) and two marine samples that were from the kelp forest and used in an experimental manipulation (kelp tanks 1 and 3), sequenced by the class in 2010. **Table S6.** Class reports for Spring 2010, showing that the students covered a large range of topics and learned about many characteristics of genomic data. Click here for file
