# Supplementary material for: Microbes, metagenomes and marine mammals: enabling the next generation of scientist to enter the genomic era
Source: BMC Genomics. 2013 Sep 4;14:600. doi: 10.1186/1471-2164-14-600 (PMC3766688; doi:10.1186/1471-2164-14-600)
Supplement: Additional file 1: Table S1 — Lecture and lab schedule for the ecological metagenomics class. Table S2. The question for the Pre and Post quiz given to students in the ecological metagenomics class. Table S3. The proportion of repeat regions identified in the California sea lion, panda, dog, human, and mouse. Table S4. The number of sequences that met each of the filter controls on three sequencing runs conducted by the students on a titanium plate divided into 4 lanes. Table S5. The sequence characteristics of three metagenomes, constructed from the surface water off Mission Beach (California) and two marine samples that were from the kelp forest and used in an experimental manipulation (kelp tanks 1 and 3), sequenced by the class in 2010. Table S6. Class reports for Spring 2010, showing that the students covered a large range of topics and learned about many characteristics of genomic data. [file 1471-2164-14-600-S1.docx]

**Additional file 1**

# Microbes, metagenomes and marine mammals: enabling the next generation of scientist to enter the genomic era

### R. A. Edwards^1^, J. Matthew Haggerty^2^, Noriko Cassman^2^, Julia C. Busch^2,3^, Kristen Aguinaldo^2^, Sowmya Chinta^2^, M. Houle Vaughn^4^, Robert Morey^1^, Timothy T. Harkins ^5,6^, Clotilde Teiling^5^, K. Fredrikson^5,7^, and E. A Dinsdale^1§^

^1^ Computer Sciences Department, San Diego State University, 5500 Campanile Dr. San Diego, CA 92182, USA.

^2^ Biology Department, San Diego State University, 5500 Campanile Dr. San Diego, CA 92182, USA.

^3^ Current Address: Scripps Institute of Oceanography, University of California, San Diego, 9500 Gilman Drive, La Jolla 92023, USA

^4^ School of Teacher Education, San Diego State University, 5500 Campanile Dr. San Diego, CA 92182, USA.

^5^ Roche 454 Lifesciences, 15 Commercial Street, Branford, CT 06405 USA

^6^ Current Address: Life Technologies, Advanced Application Development, Beverly, MA 01915, USA

^7^ Current Address: Immun Array 800, East Leigh Street, Suite 15, Richmond, VA 23219

**Additional file 1: Table S1.**

Lecture and lab schedule for the ecological metagenomics class. Lecture topics and suggested reading are also given. The students are expected to present on paper during the semester.

| **Week** | **Lecture/ reading list** | **Practical** | **Notes and Comments** |
| --- | --- | --- | --- |
| 1 | Course introduction, goals, and aims. Overview of high throughput sequencing technology and its impact on our future [[1](#_ENREF_1), [2](#_ENREF_2)]. | Introduce the sequencer, basic skills, pipetting, magnetic separation,  Dilutions, serial dilutions  Plating Bacteria | Water and organism associated samples will be collected for the students to extract metagenomic DNA. Individual microbes will be grown for genomic DNA. Students will have to re-streak plates during the week to obtain enough DNA from the one genome to sequence. Plating will be conducted on TCBS plates to obtain Vibrios, which are an important microbe in the marine environment. DNA extraction kits will be required |
| 2 | Review of pyrosequencing technology [[3](#_ENREF_3), [4](#_ENREF_4)]. | Extract DNA  Demonstration with TFF | Metagenomics samples will be collected from the marine environment and the water will be brought back to the lab filtered as a demonstration. |
| 3 | Comparisons of sequencing technologies [[5-7](#_ENREF_5)] | Quantify DNA | Quantification uses pico green and provides experience with standard curves |
| 4 | Metagenomics – why (part 1). Comparison of traditional methods with new sequencing technology [[8](#_ENREF_8), [9](#_ENREF_9)] | Module 1) Rapid Library preparation | Taught as a whole class  Lab book hand in |
| 5 | Metagenomics of coral and coral reef water [[10](#_ENREF_10), [11](#_ENREF_11)] | Quantify rapid libraries | DNA libraries are quantified using the bioanalyzer to identify the length of the DNA and a standard curve to determine the amount of DNA |
| 6 | Metagenomics of the marine environment, both microbial and viral [[12](#_ENREF_12), [13](#_ENREF_13)] | Module 2) breaking the emulsion  Module 3) emPCR  Module 4) load the plate  Module 5) run the sequencer | The rotation starts here. The groups of students will conduct one of the four processes. |
| 7 | Metagenomic – human gut [[14](#_ENREF_14), [15](#_ENREF_15)] | Module 2) breaking the emulsion  Module 3) emPCR  Module 4) load the plate  Module 5) run the sequencer | Lab book hand in |
| 8 | Metagenomics of extreme environments [[16](#_ENREF_16), [17](#_ENREF_17)] | Module 2) breaking the emulsion  Module 3) emPCR  Module 4) load the plate  Module 5) run the sequencer |  |
| 9 | Metagenomics – functional annotations [[18](#_ENREF_18)] | Module 2) breaking the emulsion  Module 3) emPCR  Module 4) load the plate  Module 5) run the sequencer |  |
| 10 | Metagenomics - Insect related genomic research [[19](#_ENREF_19), [20](#_ENREF_20)] | Module 6) Enrichment |  |
| 11 | Eukaryotic genomes -  Human and Neanderthal genome [[21](#_ENREF_21), [22](#_ENREF_22)] | Review sequencing output from the Instrument | Complete instrument quiz  Lab book hand in. |
| 12 | Panda and dog genome - [[23](#_ENREF_23), [24](#_ENREF_24)] | Module 7) Annotation of sequences - Genomic annotation via SEED | This will require access to a computer lab. The students will write a report describing the gene content, function and/or taxonomic make-up and ecological relevance of a genomes or metagenomes. |
| 13 | Comparative genomics: investigating the arrangement of the DNA between organism and inferring it genetic potential [[25](#_ENREF_25), [26](#_ENREF_26)] | Module 7) Annotation of sequences  Metagenomic annotation using MG-RAST | This will require access to a computer lab |
| 14 | Comparative genomics: Archaea [[27](#_ENREF_27), [28](#_ENREF_28)] | Module 7) Annotation of sequences  Eukaryotic annotation using Repeat Masker, NCBI, genescan | A few contigs of the Sea lion genome will be provided to the students to explore the aspects of Eukaryotic genomes. This will require access to a computer lab |
| 15 | Bacterial genome comparisons: [[29](#_ENREF_29), [30](#_ENREF_30)] | Annotation and Analysis  Time available for own analysis | Lab book hand in |
| Finals week | Hand in report |  |  |

**Additional file 1: Table S2.**

The question for the Pre and Post quiz given to students in the ecological metagenomics class.

1. Describe the structure of DNA in a diagram or short paragraph.

2. Name the four nucleotides.

3. Describe how to use a micro-pipette?

4. How long did it take to sequence the human genome and how much did it cost?

5. Give two examples of how DNA sequences can be used?

6. Describe how pyrosequencing works.

7. Once DNA sequences are obtained, what is a process used to annotate it?

8. How do sequencing microbial and viral communities help in describing their ecology?

9a. How many letters in a codon?

9b. How many codons are there?

9c. What is the start codon?

10. What are the three domains of life?

**Additional file 1 Table S3.**

The proportion of repeat regions identified in the California sea lion panda, dog, human, and mouse.

| **Repeat type** | **Sea lion** | **Panda [24]** | **Dog [23]** | **Human [21]** | **Mouse [31]** |
| --- | --- | --- | --- | --- | --- |
| Lines  Line 1  Line 2  Line 3 | 19.47  16.40  2.7  0.28 | 18.2 | 16.49  14.5  1.84  0.15 | 21.61  17.93  3.36  0.32 | 17.36  16.99  0.34  0.04 |
| Sine  Lts  B1 (Alu)  B2  B4  ID  MIR | 6.94    0.0        2.44 | 7.9 | 9.12  7.44          1.84 | 13.95    11.00        2.95 | 7.45    2.42  2.15  2.17  0.2  0.51 |
| LTR  ERV1  ERVK  ERVL  MalR | 5.18  0.08  0.96  1.73  2.28 | 5.6 | 3.25  0.58  0.0  0.95  1.75 | 8.88  3.09  0.32  1.59  3.87 | 8.92  0.61  2.84  0.09  4.35 |
| DNA  MER1 Type  MER2 type  Tip 100  AcHobo  Mariner  Tc2 | 2.96 | 3.2 | 1.88  1.08  0.39  0.02  0.20  0.02  0.05 | 3.09  1.41  1.09  0.15  0.15  0.10  0.05 | 0.78  0.56  0.15  0.03  0.02  0.01  0.01 |
| Unclassified | 0.06 | 0.1 | 0.1 | 0.01 | 0.32 |
| Total repeats | 34.75 | 36.2 | 30.75 | 47.68 | 34.84 |

**Additional file 1: Table S4.**

The number of sequences that met each of the filter controls on three sequencing runs conducted by the students on a titanium plate divided into 4 lanes. A key pass is a well that has a bead with either sample DNA or control DNA. The “key” refers to a DNA tag that is recognized by the instrument software and used in the sequence processing. A dot bead is a bead that has no DNA on it. A mixed bead is a bead that has two DNA templates. Sequences that are too short will be removed by the short quality filter and bead that only consists of primer sequence will be removed by the short primer filter. These filters are built into the sequencing software.

| **Description of run** | **Key**  **Pass** | **Dot**  **Beads** | **Mixed Beads** | **Short Quality** | **Short Primer** | **Pass**  **Filter** |
| --- | --- | --- | --- | --- | --- | --- |
| SeaLion04 & Pseudomonas02 (Genome) |  |  |  |  |  |  |
| Lane 1 | 488565 | 19784 | 43894 | 156232 | 221 | 268434 |
| Lane 2 | 465172 | 20949 | 38904 | 135427 | 304 | 269588 |
| Lane 3 | 477720 | 24376 | 83513 | 204565 | 455 | 164811 |
| Lane 4 | 448731 | 17011 | 83122 | 188285 | 357 | 159956 |
| Average | 470047 | 20530 | 62358.2 | 171127.3 | 334.2 | 215697.3 |
| SeaLion07 |  |  |  |  |  |  |
| Lane 1 | 451973 | 16218 | 38845 | 147360 | 133 | 249417 |
| Lane 2 | 434458 | 15835 | 31936 | 120446 | 117 | 266124 |
| Lane 3 | 443035 | 17728 | 24693 | 125005 | 140 | 275469 |
| Lane 4 | 430354 | 18034 | 28767 | 136246 | 147 | 247160 |
| Average | 439955 | 16953.7 | 31060.2 | 132264.3 | 134.2 | 259542.5 |
| Kelp bacteria 9 & 11 genome Pab5 / Brazil Cal2 Metagenome |  |  |  |  |  |  |
| Lane 1 | 443441 | 71214 | 124131 | 65678 | 287 | 182131 |
| Lane 2 | 444249 | 51945 | 111486 | 59456 | 43 | 221319 |
| Lane 3 | 475877 | 87133 | 95602 | 65639 | 88 | 227415 |
| Lane 4 | 497389 | 71750 | 208905 | 76884 | 165 | 139685 |
| Average | 465239 | 70510.5 | 135031 | 66914.2 | 145.7 | 192637.5 |

**Additional file 1: Table S5.**

The sequence characteristics of three metagenomes, constructed from the surface water off Mission Beach (California) and two marine samples that were from the kelp forest and used in an experimental manipulation (kelp tanks 1 and 3), sequenced by the class in 2010. The sample from Malden in the central Pacific was collected by Dinsdale and sequenced externally. The number and length of these metagenomes provided an appropriate amount of data for describing microbial communities. The number of sequences showing similarity to microbial taxa and functional genes identify by the students was typical of a metagenome prepared and sequenced in a sequencing facility. The number of sequences showing similarity to the human genome was low suggesting that human contamination did not occur.

| **Characteristics** | **Mission Beach** | **Kelp tank 1** | **Kelp tank 3** | **Malden** |
| --- | --- | --- | --- | --- |
| Number of sequences | 95,709 | 107,833 | 136,192 | 48,258 |
| Average length (bp) | 327 | 353 | 346 | 349 |
| Number of functional similarities | 23,733 | 54,422 | 72,765 | 12,691 |
| Number of taxonomic similarities | 36,171 | 77,818 | 105,713 | 12,662 |
| Number of sequences similar to Bacteria | 30,305 | 76,653 | 104,446 | 10,863 |
| Number of sequences similar to humans | 114 | 5 | 9 | 52 |

**Additional file 1: Table S6.**

Class reports for Spring 2010, showing that the students covered a large range of topics and learned about many characteristics of genomic data. All sequences were generated by the class and represented projects being conducted in the Edwards and Dinsdale labs.

| **Title of project** | **Sequences examined (bp)** | **Summary of analysis** |
| --- | --- | --- |
| Investigating the physiological properties of the marine sample of a yellow *Staphylococcus* | 2,531,105 | Conducted a comparative analysis of the Urea cycle and identified several transposons |
| Sequencing the Sea lion genome | 15,507 | Identified genes on two contigs of the sea lion. The mitochondria was compared to NCBI database and was a 100 % match to *Zalophus californianus* |
| Genomic analysis and characterization of *Staphylococcus* yellow | 2,531,105 | Conducted a comparison of virulence genes with all known Staphylococcus and found it was lacking several virulence genes. |
| Genomic analysis of the newly discovered *Pseudomonas* | 5,204,818 | Focused on energy pathways, particularly the TCA cycle |
| Lifestyles of Viruses | 86,543,251 | Comparisons of viruses from the 4 oxygen minimum zone metagenomes |
| Bacterial genomes associated with kelp | 125,964,572 | Comparative analysis of the suppression of copper gene in bacteria from the kelp forest |
| *Salmonella enterica* serovar Enteritidis | 4,942,195 | Identified phage and explored the preprotein translocase SecY mechanisms |
| Using New sequencing technology to discover phylogenetic relationships between certain taxon | 79,552,830 | Use of g-compus to compare several contigs of the sea lion to the human genomes. |
| Salmonella: two newly sequenced strains and their relevance to existing Salmonella knowledge | 9,995,432 | Compared the core and variable genes in *Salmonella* to other previously sequenced *Salmonella* genomes. |
| Use of the genome sequencer FLX instrument to study cadmium, zinc, and cobalt resistance in two microbial communities | 77,742,168 | Compared heavy metal resistance genes across two locations and found one sample were overrepresented in these genes. |
| Comparative analysis of metagenomes from three unique marine systems | 31,293,125 | Compared metabolic functions across coral reefs, kelp forests in Southern California and Sargasso Sea metagenomes, to the nutrient availability and found distinctive differences. |
| Comparative metagenomics of incubated waters surrounding *Macrocystis pyrifera* | 169,127,001 | Compared four metagenomes that had been subjected to different levels of carbon dioxide and found that virulence genes increased with increasing carbon dioxide. |
| Yellow bacterium | 2,531,105 | Studied antibiotic resistance and toxicity compounds in all *Staphylococcus* species |
| Metabolic analysis of Pseudomonas genomes from kelp forests | 5,204,818 | Compared the ion transport and siderophores found in several *Pseudomonas* species. |
| Diversity and functional profile of bacterial communities from Abrolhos Banks Brazil |  | Compared the phylogeny and potential function of 8 metagenomes across coral reefs with varying levels of fishing |
| 454 Pyrosequencing and genome analysis of Vibrio species isolated from Pacific coast *Macrocystis* | 10,828,392 | Compared the motility and chemotaxis of *Vibrio* genomes and the sequenced genome from the kelp forest lacked features found in human pathogenic strains. |
| Whole genome analysis of *Pseudomonas* | 5,204,818 | Examined RNA and metabolic function of this genome |
| DNA metabolism in a Kelp genome | 10,729,741 | Describe DNA repair in a new genome with particular focus on the RecA and RecR genes |
| Low-coverage genomic sequencing of California sea lion *Zalophus californianus*. | 3,600,000 | Conducted an analysis of the repeat regions, mitochondria and genes present in the newly sequenced sea lion genome. |
| Total | 665,941,983 |  |

**References**

1. Collins FS: **Genome research: the next generation.** *Cold Spring Harb Symp Quant Biol* 2003, **68:**49-54.

2. Collins FS, Green ED, Guttmacher AE, Guyer MS: **A vision for the future of genomics research.** *Nature* 2003, **422:**835-847.

3. Ronaghi M, Uhlen M, Nyren P: **A sequencing method based on real-time pyrophosphate.** *Science* 1998, **281:**363, 365.

4. Rothberg JM, Leamon JH: **The development and impact of 454 sequencing.** *Nat Biotechnol* 2008, **26:**1117-1124.

5. Huse SM, Huber JA, Morrison HG, Sogin ML, Welch DM: **Accuracy and quality of massively parallel DNA pyrosequencing.** *Genome Biol* 2007, **8:**R143.

6. Metzker ML: **Sequencing technologies - the next generation.** *Nat Rev Genet* 2010, **11:**31-46.

7. Metzker ML: **Applications of next-generation sequencing technologies - the Next Generation.** *Nat Rev Genet* 2010, **11:**31-46.

8. DeLong EF, Karl DM: **Genomic perspectives in microbial oceanography.** *Nature* 2005, **437:**336-342.

9. Hugenholtz P, Tyson GW: **Microbiology - Metagenomics.** *Nature* 2008, **455:**481-483.

10. Dinsdale EA, Pantos O, Smriga S, Edwards RA, Wegley L, Angly F, Brown E, Haynes M, Krause L, Sala E, et al: **Microbial ecology of four coral atolls in the northern Line Islands.** *Plos One* 2008, **3:**e1584.

11. Wegley L, Edwards RA, Rodriguez-Brito B, Liu H, Rohwer F: **Metagenomic analysis of the microbial community associated with the coral *Porites astreoides*.** *Environ Microbiol* 2007, **9:**2707-2719.

12. Angly F, Felts B, Breibart M, Salamon P, Edwards RA, Carlson CA, Chan AM, Hayes R, Kelley S, Liu H, et al: **The marine viromes of four oceanic regions.** *PLoS Biol* 2006, **4:**e368.

13. Venter JC, Remington K, Heidelberg JF, Halpern AL, Rusch D, A. EJ, Wu D, Paulsen I, Nelson KE, Nelson W, et al: **Environmental genome shotgun sequencing of the Sargasso Sea.** *Science* 2004, **304:**66-74.

14. Turnbaugh PJ, Baeckhed F, Fulton L, Gordon JI: **Diet-induced obesity is linked to marked but reversible alterations in the mouse distal gut microbiome.** *Cell Host & Microbe* 2008, **3:**213-223.

15. Turnbaugh PJ, Ley RE, Mahowald MA, Magrini V, Mardis ER, Gordon JI: **An obesity-associated gut microbiome with increased capacity for energy harvest.** *Nature* 2006, **444:**1027-1031.

16. Tyson GW, Chapman J, Hugenholtz P, Allen EE, Ram RJ, Richardson PM, Solovyev VV, Rubin EM, Rokhsar DS, Banfield JF: **Community structure and metabolism through reconstruction of microbial genomes from the environment.** *Nature* 2004, **428:**37-43.

17. Edwards RA, Rodriguez-Brito B, Wegley L, Haynes M, Breitbart M, Peterson DM, Saar MO, Alexander S, Alexander EC, Rohwer F: **Using pyrosequencing to shed light on deep mine microbial ecology.** *Bmc Genomics* 2006, **7**.

18. Dinsdale EA, Edwards RA, Hall D, Angly F, Breitbart M, Brulc JM, Furlan M, Desnues C, Haynes M, Li LL, et al: **Functional metagenomic profiling of nine biomes.** *Nature* 2008, **452:**629-632.

19. Scott JJ, Budsberg KJ, Suen G, Wixon DL, Balser TC, Currie CR: **Microbial community structure of leaf-cutter ant fungus gardens and refuse dumps.** *PloS One* 2010, **5**.

20. Oliver K, Degnan P, Hunter M, Moran N: **Bacteriophages encode factors required for protection in a symbiotic mutualism** *Science* 2009, **325:**992-994.

21. de Jong P, Catanese JJ, Osoegawa K, Shizuya H, Choi S, Chen YJ, Cons IHGS: **Initial sequencing and analysis of the human genome.**  *Nature* 2001, **412:**565-566.

22. Green RE, Krause J, Briggs AW, Maricic T, Stenzel U, Kircher M, Patterson N, Li H, Zhai WW, Fritz MHY, et al: **A Draft Sequence of the Neandertal Genome.** *Science* 2010, **328:**710-722.

23. Kirkness EF, Bafna V, Halpern AL, Levy S, Remington K, Rusch DB, Delcher AL, Pop M, Wang W, Fraser CM, Venter JC: **The dog genome: Survey sequencing and comparative analysis.** *Science* 2003, **301:**1898-1903.

24. Li RQ, Fan W, Tian G, Zhu HM, He L, Cai J, Huang QF, Cai QL, Li B, Bai YQ, et al: **The sequence and *de novo* assembly of the giant panda genome.** *Nature* 2010, **463:**311-317.

25. Lee DS, Burd H, Liu J, Almaas E, Wiest O, Barabasi AL, Oltvai ZN, Kapatral V: **Comparative genome-scale metabolic reconstruction and flux balance analysis of multiple *Staphylococcus aureus* genomes identify novel antimicrobial drug targets.** *J Bacteriol* 2009, **191:**4015-4024.

26. Sabbagh SC, Forest CG, Lepage C, Leclerc JM, Daigle F: **So similar, yet so different: uncovering distinctive features in the genomes of *Salmonella enterica* serovars Typhimurium and Typhi.** *FEMS Microbiol Lett* 2010, **305:**1-13.

27. Makarova KS, Koonin EV: **Evolutionary and functional genomics of the Archaea.** *Cur Opinion Microbiol* 2005, **8:**586-594.

28. Falb M, Mueller K, Koenigsmaier L, Oberwinkler T, Horn P, von Gronau S, Gonzalez O, Pfeiffer F, Bornberg-Bauer E, Oesterhelt D: **Metabolism of *halophilic archaea*.** *Extremophiles* 2008, **12:**177-196.

29. Hasan NA, Grim CJ, Haley BJ, Chun J, Alam M, Taviani E, Hoq M, Munk AC, Saunders E, Brettin TS, et al: **Comparative genomics of clinical and environmental *Vibrio mimicus*.** *PNAS* 2010, **107:**21134-21139.

30. Miller WG, Parker CT, Rubenfield M, Mendz GL, Wosten MMSM, Ussery DW, Stolz JF, Binnewies TT, Hallin PF, Wang GL, et al: **The complete genome sequence and analysis of the Epsilonproteobacterium *Arcobacter butzleri*.** *Plos One* 2007, **2**.

31. Waterston RH, Lindblad-Toh K, Birney E, Rogers J, Abril JF, Agarwal P, Agarwala R, Ainscough R, Alexandersson M, An P *et al*: **Initial sequencing and comparative analysis of the mouse genome**. *Nature* 2002, **420**:520-562.
